# Factors Associated with Medication Adherence among Patients with Schizophrenia in Mekelle, Northern Ethiopia

**DOI:** 10.1371/journal.pone.0120560

**Published:** 2015-03-27

**Authors:** Tadele Eticha, Amha Teklu, Dagim Ali, Gebremedhin Solomon, Adissu Alemayehu

**Affiliations:** Department of Pharmacy, College of Health Sciences, Mekelle University, Mekelle, Ethiopia; Federal University of Rio de Janeiro, BRAZIL

## Abstract

**Background:**

Non-adherence to antipsychotic medication has a negative impact on the course of illness resulting in increased risk of relapse, rehospitalization and suicide, and increased costs to healthcare systems. The objective of this study was to investigate factors associated with medication adherence among patients with schizophrenia at Ayder Referral Hospital and Mekelle Hospital in Mekelle, Tigray region, Northern Ethiopia.

**Methods:**

The study was a cross-sectional survey in which sociodemographic characteristics, drug attitudes, insight and side effects were measured and explored in terms of their relationship with medication adherence. A structured questionnaire as a data collection tool was used. Data were analyzed with the help of SPSS Version 20.0.

**Results:**

A total of 393 patients participated, 26.5% were non-adherent to their antipsychotic medication. The factors significantly associated with better adherence were positive treatment attitudes (AOR = 1.40, 95% CI: 1.26, 1.55), fewer side effects (AOR = 0.97, 95% CI: 0.94, 0.99), awareness of illness (AOR = 1.44, 95% CI: 1.12, 1.85) and the ability to relabel symptoms (AOR = 1.57, 95% CI: 1.19, 2.07). However, khat chewers (AOR = 0.24, 95% CI: 0.09, 0.68), being illiterate (AOR = 0.13, 95% CI: 0.03, 0.47) and older age group (AOR = 0.03, 95% CI: 0.01, 0.16) were associated with less medication adherence.

**Conclusions:**

A high prevalence of medication non-adherence was found among patients with schizophrenia. Intervention strategies focused on educating the patients to better understand the illness, medications and their potential side effects might be useful in improving adherence to antipsychotic medication treatment.

## Introduction

Antipsychotic medication adherence plays a key role in patients with schizophrenia, and regular treatment has been proven to ameliorate symptoms and reduce relapse rates [1]. However, treatment non-adherence remains one of the greatest challenges in psychiatry [2]. A comprehensive review [3] reported that the rate of medication non-adherence in patients with schizophrenia is as high as 40%–50%.

Non-adherence to antipsychotic medication has a negative impact on the course of illness resulting in consequences to patients, society and healthcare systems. Many studies [4–6] investigated that hospitalization rates were significantly higher among non-adherent patients compared with adherent ones. Although there was heterogeneity in the definition of adherence and measures of adherence used, a consistent connection between lower adherence rates and higher hospitalization risk has been revealed. A systematic review [7] checked suicide rates out because of non-adherence and reported a trend where non-adherence to medication treatment was associated with a significant increase in the risk of suicide. Non-adherence to antipsychotic medication was related to exacerbation of psychotic symptoms, increased aggression and worse prognosis which may result in resistance to drugs and to the development of chronic psychotic symptoms. It was investigated that non-adherence was also significantly related to violence (i.e non-adherent patients were more violent than adherent patients). Non-adherence to medication can lead to relapse, which can mean more visits to the emergency room, rehospitalizations and increased need for clinician intervention—all of which lead to increased costs to healthcare systems [7,8].

The factors consistently associated with non-adherence in patients with schizophrenia are lack of insight, attitudes towards their illness and the medication, past experiences with their illness and its treatment, substance abuse, adverse drug reactions and lack of social support [7,9,10]. However, sociodemographic factors of the patients are not consistent predictors of poor adherence [7,9].

A qualitative study from rural Ethiopia explored the reasons for non-adherence to antipsychotic medications in people with schizophrenia from the perspectives of patients, their caregivers, health professionals and research field workers. Many of the factors associated with non-adherence include inadequate availability of food, lack of family/social support, lack of insight, failure to improve with treatment, medication side effects, substance abuse, stigma and dissatisfaction with the attitude of health care providers [11].

Therefore, identifying the predictors of non-adherence is the first step to design suitable intervention strategies aimed at preventing or reducing the negative consequences of non-adherence [12]. Interventions targeted specifically to problems of non-adherence were more likely to be effective than were more broadly based treatment interventions [13]. The aims of the present study were to determine non-adherence rate and factors related to antipsychotic medication adherence among patients with schizophrenia which could suggest means for improving adherence in these people.

## Materials and Methods

### Setting and study design

A cross-sectional study was undertaken from April to May 2014 at Ayder Referral Hospital and Mekelle Hospital in Mekelle. Mekelle is the capital city of the Tigray Region and located at 783 kilometers to the north of Addis Ababa, the capital city of Ethiopia. Both hospitals provide a broad range of medical services to populations in its catchment areas of the Tigray, Afar and southeastern parts of the Amhara Regional States. Moreover, Ayder Referral Hospital is a teaching hospital in College of Health Sciences, Mekelle University.

### Study sample and procedures

Consecutive schizophrenic patients visited Ayder Referral Hospital and Mekelle Hospital were asked to participate in the study. Patients were screened based on their ability to understand the relevant information, appreciate a situation and its consequences, and reason rationally by the clinicians to identify suitable patients meeting the following inclusion criteria: aged between18 and 65 years of age; a diagnosis of schizophrenia; capacity to give informed consent; and continuous therapy at least for three months before the study. Exclusion criteria were co-morbid mood disorder, serious medical condition and mental retardation. The study was approved by the Health Research Ethics Review Committee of College of Health Sciences, Mekelle University. The participants were clearly informed that participation was voluntary and information obtained would be anonymous and confidential. Then written informed consent was obtained from patients who agreed to participate.

The sample size was determined using the formula for a single population proportion for cross-sectional study, based on the non-adherence proportion of 50% (to achieve maximum representative sample size) and 5% margin of error at 95% confidence level. The total sample size calculated was 403 after considering 5% non-response rate.

### Data collection

Study participants were interviewed by using a structured questionnaire composed of a variety of assessment methods. The questionnaire was originally developed in English translated into the local language (Tigrigna) and back translated to English to check the accuracy. The questionnaire consists of patient background variables, insight, beliefs about treatment, psychiatric medication adherence, side effects and satisfaction with medication.

#### Patient background variables

The survey included socio-demographic characteristics and alcohol/cigarette/khat use. Socio-demographic characteristics contained: age, gender, ethnicity, marital status, religion status, education status, residence, employment status and income. Substance use (alcohol, cigarette and/or khat) within three months before the survey was also investigated.

#### Insight

The three areas of insight, insight into the need for treatment, awareness of illness and the ability to relabel experiences, were measured using the Insight Scale for Psychosis (ISP). ISP is an 8-item self-report scale that has been shown to have good reliability and validity in people who experience psychotic symptoms. The score of each subscale ranges from 0 to 4 [14].

#### Beliefs about treatment

Drug Attitude Inventory (DAI), a 10-item true/false scale, was employed to assess beliefs about treatment. A correct answer scored as +1, and an incorrect answer scored as −1. The final DAI score is the sum of the pluses and minuses. A positive total score indicates a positive attitude towards medication, and a negative total score, a negative attitude [15].

#### Psychiatric medication adherence

Adherence was measured using a modified version of the Medication Adherence Rating Scale (MARS), which is a 10-item self-report scale. The items are answerable by a yes/no response, with corresponding 0 and 1 value, respectively. A MARS score equal to 3 or above indicates adherence, and non-adherence is defined as scores less than or equal to 2 [16].

#### Side effects

Medication side effects were measured by presenting subjects with a list of 16 items (e.g., sleep problems; shaking/tremors; restlessness/jitteriness) with responses that ranged from “not at all” to “very much” [17].

#### Satisfaction with medication

One question asked: “How satisfied were you with the psychiatric medications you were taking before you came to this program?” The responses range from “not at all satisfied” to “very satisfied” [18].

### Data Analysis

The data were entered and analysed using the Statistical Program for Social Sciences (SPSS) version 20.0 for Windows. Descriptive statistics such as frequencies and proportions were used for data summarization. Bivariate logistic regression analysis was performed to determine the association between each of the independent variables and adherence using cross tabulations and logistic regression. Factors significantly associated with adherence in the bivariate analysis were considered for multivariate logistic regression analysis. The level of significance for independent variables was set at 0.05 (two-sided).

## Results

### Sociodemographic characteristics of the study participants

Out of the total 403 study participants, 393 participants filled the questionnaire completely which gave a response rate of 97.5%. Sociodemographic characteristics of the respondents and substance use are summarized in Table 1. The mean age of the respondents was 30.52, with the majority (48.1%) being between 25 to 34 years. The majority (72%) of the respondents were males. More than 90% of the study participants were Tigre by ethnicity and 61.1% of them were Orthodox by religion. More than half (57.3%) of the study participants were unmarried. The majority of (48.9%) of the patients had tertiary education. About two third of the patients were alcohol consumers (67.9%) while about haft (49.1%) were smokers.

**Table 1:** Socio-demographic characteristics of study participants.

**Characteristics**		**Frequency**	**Percent**
**Age group**	18–24	100	25.4
	25–34	189	48.1
	35–44	72	18.3
	45–64	32	8.1
**Gender**	Male	283	72
	Female	110	28
**Ethnic group**	Tigre	363	92.4
	Amhara	17	4.3
	Other	13	3.3
**Marital status**	Unmarried	225	57.3
	Married	93	23.7
	Divorced	32	8.1
	Separated	31	7.9
	Widowed	12	3.1
**Religion**	Orthodox	240	61.1
	Catholic	32	8.1
	Protestant	33	8.4
	Muslim	88	22.4
**Education status**	Illiterate	28	7.1
	Primary	60	15.3
	Secondary	113	28.8
	Tertiary	192	48.9
**Residence**	Urban	173	44.0
	Rural	220	56.0
**Employment status**	Unemployed	151	38.4
	Employed	162	41.2
	Student	80	20.4
**Income per month (ETB)**	<250	156	39.7
	250–500	52	13.2
	≥500	185	47.1
**Alcohol consumption**	Yes	267	67.9
	No	126	32.1
**Smoking cigarette**	Yes	193	49.1
	No	200	50.9
**Chewing chat**	Yes	148	37.7
	No	245	62.3

Exchange rate: 1 USD = 19.82 Ethiopian Birr (ETB).

### Prevalence of adherence

The prevalence of non-adherence, defined as a MARS total score of less than or equal to 2, among the study participants was (n = 393, 26.5%). The majority (73.1%) of the non-adherent patients were males.

### Factors associated with antipsychotic medication adherence

Table 2 shows the association of sociodemographic factors with antipsychotic medication adherence among patients with schizophrenia. The following variables were significantly related to adherence in bivariate analysis: age, marital status, education status, residence, employment status, alcohol consumption, cigarette smoking and khat chewing. Table 3 indicates the association of variables such as treatment attitudes, medication side effects, satisfaction with medication, and insight with adherence. Attitudes toward the treatment, side effects, areas of insight like the ability to relabel symptoms and awareness of illness were associated with medication adherence.

**Table 2:** Association of sociodemographic factors with antipsychotic medication adherence among patients with schizophrenia.

**Variables**		**Adherent, n(%)**	**Non-adherent, n(%)**	**OR (95% CI)**	**p-value**
**Age group**	18–24	82(82)	18(18)	1	
	25–34	148(78.3)	41(21.7)	0.79(0.43, 1.47)	0.459
	35–44	50(69.4)	22(30.6)	0.50(0.24, 1.02)	0.057
	45–64	9(28.1)	23(71.9)	0.09(0.03, 0.23)	0.000
**Gender**	Male	207(73.1)	76(26.9)	1	
	Female	82(74.5)	28(25.5)	1.08(0.65, 1.78)	0.778
**Ethnic group**	Tigre	265(73)	98(27)	1	
	Amharic	15(88.2)	2(11.8)	2.77(0.62, 12.35)	0.181
	Other	9(69.2)	4(30.8)	0.83(0.25, 2.76)	0.764
**Marital status**	Unmarried	172(76.4)	53(23.6)	1	
	Married	72(77.4)	21(22.6)	1.06(0.59, 1.88)	0.852
	Divorced	25(78.1)	7(21.9)	1.10(0.45, 2.69)	0.834
	Separated	14(45.2)	17(54.8)	0.25(0.12, 0.55)	0.000
	Widowed	6(50)	6(50)	0.31(0.10, 0.99)	0.049
**Religion**	Orthodox	180(75)	60(25)	1	
	Catholic	20(62.5)	12(37.5)	0.56(0.26, 1.20)	0.136
	Protestant	28(84.8)	5(15.2)	1.87(0.69, 5.05)	0.219
	Muslim	61(69.3)	27(30.7)	0.75(0.44, 1.29)	0.303
**Education status**	Tertiary	42(21.9)	150((78.1)	1	
	Secondary	80(70.8)	33(29.2)	0.15(0.40, 1.15)	0.152
	Primary	50(83.3)	10(16.7)	1.40(0.66, 2.99)	0.386
	Illiterate	9(32.1)	19(67.9)	0.13(0.06, 0.32)	0.000
**Residence**	Urban	116(67.1)	57(32.9)	1	
	Rural	173(78.6)	47(21.4)	1.81(1.15, 2.84)	0.010
**Employment status**	Unemployed	102(67.5)	49(32.5)	1	
	Employed	122(75.3)	40(24.7)	1.47(0.89, 2.40)	0.129
	Student	65(81.2)	15(18.8)	2.08(1.08, 4.01)	0.029
**Income per month (ETB)**	<250	111(71.2)	45(28.8)	0.77(0.47, 1.25)	0.290
	250–500	37(71.2)	15(28.8)	0.77(0.38, 1.53)	0.456
	≥500	141(76.2)	44(23.8)	1	
**Alcohol consumption**	No	103(81.7)	23(18.3)	1	
	Yes	186(69.7)	81(30.3)	0.51(0.30, 0.86)	0.012
**Cigarette smoking**	No	161(80.5)	39(19.5)	1	
	Yes	128(66.3)	65(33.7)	0.48(0.30, 0.76)	0.002
**Khat chewing**	No	195(79.6)	50(20.4)	1	
	Yes	94(63.5)	54(36.5)	0.45(0.28, 0.71)	0.001

**Table 3:** Attitudes, medication side effects, satisfaction with medication, and insight.

**Measure**	**Adherent**	**Non adherent**	**OR (95% CI)**	**p-value**
	mean(SD)	mean(SD)		
**Treatment attitudes** ^**1**^	3.25(3.37)	−0.23(2.57)	1.38(1.27, 1.50)	0.000
**Medication side effects** ^**2**^	22.88(12.54)	30.91(14.12)	0.95(0.94, 0.97)	0.000
**Satisfaction with medication** ^**3**^	2.94(1.43)	2.53(1.61)	1.20(1.04, 1.38)	0.015
**Insight**				
**Relabel symptoms** ^**4**^	2.25(1.22)	1.72(1.29)	1.41(1.17, 1.70)	0.000
**Awareness of illness** ^**4**^	2.32(1.40)	1.57(1.44)	1.43(1.22, 1.67)	0.000
**Accept the need for treatment** ^**4**^	2.93(1.02)	2.92(0.98)	1.02(0.81, 1.27)	0.889

Higher score indicates a

^1^more positive attitude towards treatment but negative value indicates negative attitude towards treatment,

^2^more severe side effect,

^3^more satisfaction with medication, and

^4^greater insight.

Results of multivariate logistic regression of independent variables and antipsychotic medication adherence are shown in Table 4. Factors those significantly related to adherence in bivariate analyses were included in multivariate logistic regression analysis. Findings from the multivariate analysis showed that individuals whose age group of 45–64 (AOR = 0.03, 95% CI: 0.01, 0.16) were significantly less adherent than the younger age groups. Likewise, illiterate patients (AOR = 0.13, 95% CI: 0.03, 0.47) and khat chewers (AOR = 0.24, 95% CI: 0.09, 0.68) were significantly associated with less adherence compared to their counterparts. Nevertheless, sociodemographic characteristics such as marital status, residence, employment status, alcohol consumption and cigarette smoking were retained in the multivariate analysis as confounders of independent predictors of adherence.

**Table 4:** Results of multivariate logistic regression of independent variables and antipsychotic medication adherence.

**Variables**		**AOR** ^1^ **(95% CI)**	**p-value**
**Age group**	18–24	1	
	25–34	1.42(0.58, 3.48)	0.445
	35–44	0.85(0.26, 2.84)	0.794
	45–64	0.03(0.01, 0.16)	0.000
**Marital status**	Unmarried	1	
	Married	1.75(0.69, 4.45)	0.239
	Divorced	2.21(0.59, 8.26)	0.239
	Separated	1.30(0.39,4.32)	0.664
	Widowed	8.43(0.47, 151.66)	0.148
**Education status**	Tertiary	1	
	Secondary	0.56(0.27, 1.17)	0.123
	Primary	1.05(0.33, 3.38)	0.937
	Illiterate	0.13(0.03, 0.47)	0.002
**Residence**	Urban	1	
	Rural	1.71(0.85, 3.47)	0.135
**Employment status**	Unemployed	1	
	Employed	0.11(0.50, 1.45)	0.793
	Student	1.01(0.38, 2.69)	0.980
**Alcohol consumption**	No	1	
	Yes	1.07(0.44, 2.58)	0.882
**Cigarette smoking**	No	1	
	Yes	1.72(0.62, 4.82)	0.300
**Khat chewing**	No	1	
	Yes	0.24(0.09, 0.68)	0.007
**Treatment attitudes**		1.40(1.26, 1.55)	0.000
**Medication side effects**		0.97(0.94, 0.99)	0.010
**Satisfaction with medication**		0.99(0.81, 1.21)	0.927
**Insight**			
**Relabel symptoms**		1.57(1.19, 2.07)	0.001
**Awareness of illness**		1.44(1.12, 1.85)	0.004

^1^Adjusted for age, marital status, education status, residence, employment status, alcohol consumption, cigarette smoking, khat chewing, treatment attitudes, medication side effects, satisfaction with medication, relabel symptoms and awareness of illness.

Adherent patients had positive attitudes (a mean DAI score of 3.25) towards treatment, while non-adherent patients had negative attitudes (a mean DAI score of −0.23), indicating that positive attitudes towards treatment were related to treatment adherence (AOR = 1.40, 95% CI: 1.26, 1.55). Medication side effects were also found to be significantly associated with adherence (AOR = 0.97, 95% CI: 0.94, 0.99). Patients who had fewer side effects were more likely to be adherent to their medication treatment. Furthermore, two areas of insight: the ability to relabel symptoms (AOR = 1.57, 95% CI: 1.19, 2.07) and awareness of illness (AOR = 1.44, 95% CI: 1.12, 1.85) were observed to be statistically significant between the adherent and non-adherent groups. Patients with higher mean scores of the ability to relabel symptoms and awareness of illness were more likely to be adherent to their medication.

## Discussion

In this study, 26.5% of respondents (n = 393) were non-adherent with antipsychotic treatment. This finding is lower than the non-adherence rates reported in Southwest Ethiopia, 41.2% [19]; England, 29% [20]; Hong Kong, 30% [15] and Egypt, 74% [21]. The remarkably lower prevalence of non-adherence compared to the study conducted in Egypt could be explained by the differences in assessing medication adherence methods and sample size. One major difficulty in researching and assessing the magnitude of non-adherence in schizophrenia is the lack of consistent and agreed upon definitions of this phenomenon. For example, some patients who are considered adherent in one study could be categorized as non-adherent in another depending on where the distinction is made [10]. A10-item modified version of the MARS was used in this study, which is answerable by a yes/no response. A MARS score equal to 3 or above indicates adherence, and non-adherence is defined as scores less than or equal to 2. On the other hand, the Arabic version of the 8-item Morisky Medication Adherence Scale (MMAS) with seven yes/no questions and one question answered on a 5-point Likert scale was used in the study conducted in Egypt. According to the scoring system for the MMAS, patients who scored 8 and <8 were considered as adherent and non-adherent, respectively. In addition, the sample size employed in this survey is relatively higher (393 versus 92) than the study conducted in Egypt. Nevertheless, medication adherence was measured using the self-reported 4-item Morisky scale in Southwest Ethiopia.

A number of factors were significantly associated with non-adherence to antipsychotic medication. Older patients were found to be less adherent to their medication in this survey. However, younger patients were significantly associated with a greater risk of non-adherence to antipsychotic therapy as reported in other studies [6,15]. These differences might be related to the higher prevalence of comorbidity such as hypertension, diabetics, and other chronic diseases in older people. Hence they are often prescribed multiple treatments and pill burden could lead to non-adherence to their medications. A study conducted among psychiatric patients at a Tertiary Care Hospital, Karachi, Pakistan reported that there was a strong correlation between the presence of a co-morbidity and non-adherence. Co-morbidity is likely to increase the number of medications being taken by the patient. This increase makes the medication regimen complex for the patients to follow or incorporate into a routine [22]. Furthermore, older patients could fail to adhere to treatments because of cognitive deficit, including working-memory loss and impaired executive performance [12]. Being illiterate was significantly correlated with non-adherence, which is in agreement with the findings of other studies [23,24]. Interventions such as routine counseling, for instance pharmacists, nurse, family members, may help improve therapy adherence among high risk patients [25]. In the present work, patients who chewed khat were more likely to be non-adherent compared to those who didn’t. Many studies [23,24,26] reported that substance abuse was found to be significantly associated with non-adherence. A qualitative study conducted among persons with schizophrenia in Butajira, Ethiopia also found that alcohol drinking and khat chewing were important factors adversely affecting adherence [11].

Non-adherence is highly influenced by patient knowledge, attitudes towards their illness and the medication [9]. The drug attitudes of respondents were positive among adherent patients and negative among non-adherent patients in this survey. These confirm that positive attitudes toward the treatment have been associated with better adherence while negative ones have been related with non-adherence like other studies [12,15]. These findings indicate interventions that explore and improve patients’ attitudes towards their medication could enhance medication adherence. In the present work, adverse effects of medication were observed to be significantly related to non-adherence. The expert survey [26] rated distress associated with persistent side effects to be important contributors to adherence problems. Some specific adverse effects have more impact on adherence than other factors though the findings of patient surveys vary [9]. Patients with schizophrenia from Butajira also cited side effects of medications as a reason for non-adherence [11]. Education on medication side effects and the importance of adherence is crucial to get the benefits of medication.

Lack of insight is one of the most important contributors to non-adherence in schizophrenia [12,26–28]. Lack of insight may range from gross denial of illness and a complete rejection of the specific diagnosis to minimization and rationalization of symptoms and a lack of appreciation that medications are required to treat specific symptoms and to reduce the risk of relapse. A significant number of patients with schizophrenia have very poor or complete absence of insight into their illness; such patients are more likely to demonstrate complete rejection of the need for treatment and be chronically non-compliant [10]. Two areas of insight: awareness of illness and the ability to relabel symptoms were significantly associated with non-adherence in the present work unlike another study [15]. This suggests that therapeutic interventions designed to improve adherence to antipsychotic treatment should aim to enhance patients’ awareness of illness and the ability to relabel symptoms, rather than focus on improving awareness of the need for treatment.

In general, psychoeducation could be helpful for the improvement of adherence to medication in this population. Psychoeducation aims to educate patients or families to better understand the illness, appropriate medications and potential side effects. It targets individuals or patient groups, sometimes families, and involves counseling sessions, and/or use of written/audiovisual materials [9]. A study conducted in Hospital Bahagia Ulu Kinta reported that psychoeducation is an important tool in improving insight into illness among patients with schizophrenia. It has been also found to have a positive effect on the attitude towards medication, relapse prevention and reduction in readmission rate [29].

The present work has some limitations. The cross-sectional design of the study fails to assess patients’ adherence behaviors over time, and although this approach is helpful to investigate associations between variables, it cannot attribute cause and effect. Self-reported adherence rates have been shown to overestimate the prevalence of adherence. Another limitation of this study pertains to the fact that there was heterogeneity in the definition of adherence and methods to measure medication adherence and therefore, it was difficult to draw exact comparisons with other studies.

## Conclusions

This study indicates a self-reported non-adherence rate of 26.5% among patients with schizophrenia. Being illiterate and older age, chewing khat, treatment attitudes, side effects, relabel symptoms and awareness of the illness were the most important risk factors for non-adherence. The findings of this study imply that psychoeducation could be helpful to improve adherence to antipsychotic medication in schizophrenia.
